# Initial rivaroxaban dosing in patients with atrial fibrillation

**DOI:** 10.1002/clc.23235

**Published:** 2019-07-17

**Authors:** Kaja Ablefoni, Alexander Buchholz, Laura Ueberham, Sebastian Hilbert, Nikolaos Dagres, Daniela Husser, Gerhard Hindricks, Andreas Bollmann

**Affiliations:** ^1^ Department of Electrophysiology Heart Center Leipzig Leipzig Germany

**Keywords:** anticoagulation, atrial fibrillation, dose reduction, rivaroxaban

## Abstract

**Background:**

Rivaroxaban is a non‐vitamin K oral anticoagulant and has been approved for prevention of stroke and systemic embolism in patients with non‐valvular atrial fibrillation (AF). Current labeling recommends 20 mg once a day (q.d.) as a standard dose and a reduced dose of 15 mg q.d. in patients with renal impairment.

**Hypothesis:**

The aim of this study was to analyze the adherence to current labeling concerning initial rivaroxaban dosing and to determine whether potential lack of such adherence is medically justified.

**Methods:**

Patients with AF initiated on rivaroxaban between January 1, 2016 and January 31, 2017, were identified in the Heart Center Leipzig database. Health records were screened to identify prescribed rivaroxaban dose, presence or absence of renal impairment, patient characteristics, further dosing‐relevant diagnoses and co‐medication with antiplatelet drugs and non‐vitamin K oral anticoagulants (NOACs).

**Results:**

We identified a total of 378 consecutive patients. In 282 cases (74.6%), rivaroxaban was prescribed in a standard dose and in 96 (25.4%) in a reduced dose. Out of 96 patients receiving a reduced dose, 50 (52.1%) did not meet labeling criteria for dose reduction. In uni‐ and multivariate regression analysis, estimated glomerular filtration rate (eGFR) (odds ratio [OR] 0.34, 95% confidence interval [CI] 0.12‐0.95, *P* = .04) was the only independent predictor of rivaroxaban underdosage.

**Conclusions:**

In clinical practice, rivaroxaban dosing is frequently incoherent with labeling. In this study, rivaroxaban was often administered underdosed. Potentially inappropriate dose reduction was significantly associated with eGFR, the same factor that is used as criterion for dose adjustment.

## 1 INTRODUCTION

Atrial fibrillation (AF), with its prevalence of 3% in adults, is the most common cardiac arrhythmia worldwide.^1^ Patients with AF have an increased risk of stroke and systemic thromboembolism. The rate of ischemic stroke among these patients is 4‐ to 5‐fold higher than among those without AF.^2^ The therapy of non‐valvular AF concentrates on reducing these risks. Commonly, dose‐adjusted vitamin K antagonists (VKAs) were considered the first‐choice medication; however, the introduction of non‐vitamin K oral anticoagulants (NOACs) provided a considerable advancement in the field.

The Rivaroxaban vs Warfarin in Non‐valvular Atrial Fibrillation (ROCKET AF) trial began the clinical use of rivaroxaban—a NOAC and an oral direct factor Xa inhibitor. Its effectiveness and safety of usage was proven to be at least equal to dose‐adjusted warfarin, in spite of fixed dosing, few drug and food interactions and without the need for continual laboratory monitoring.^3^, ^4^ However, rivaroxaban's clearance is determined by renal function, which constitutes the need for dose adjustment in patients with renal impairment. The European Medicines Agency (EMA) recommends 20 mg once a day (q.d.) as a standard dose in patients with estimated glomerular filtration rate (eGFR) ≥50 mL/min and a reduced dose of 15 mg q.d. in patients with renal impairment and eGFR of 15 to 49 mL/min.^5^ These recommendations were established in accordance with a pharmacokinetic model in which the creatinine clearance measurement showed a heightened rivaroxaban exposure in correlation with decreased renal functions. Serum rivaroxaban concentrations increased significantly with a creatinine clearance <50 mL/min.^6^, ^7^

As suggested by recently registered data, rivaroxaban is commonly dosed inadequately. Such cases occurred among patients with normal as well as insufficient renal function, causing potential over‐ or underdosing.^8^, ^9^, ^10^, ^11^ Research provides information on how such instances influence drug's safety and effectiveness. Improper dose reduction may increase the risk of cardiovascular hospitalization, while lack of a decreased dose in cases with severe renal disorder may raise all‐cause mortality and the risk of bleeding.^9^, ^10^, ^11^

The aim of this study was to analyze the adherence to current labeling concerning initial rivaroxaban dosing in clinical practice and to identify factors associated with inappropriate dose reduction.

## 2 METHODS

### 2.1 Patient population

**Figure 1:**
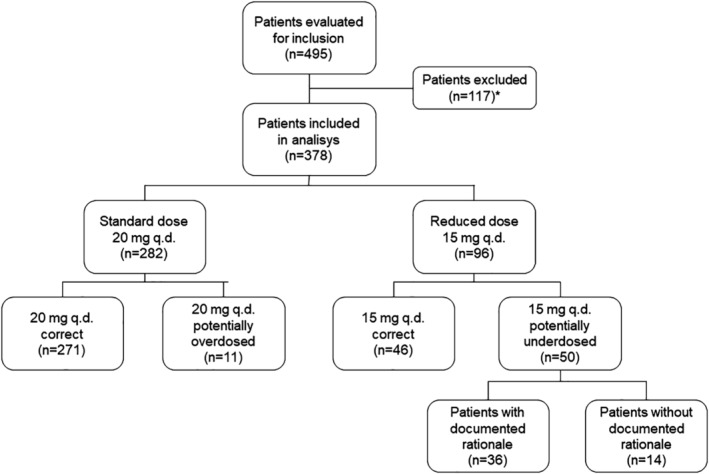
Patient selection. *Reasons for exclusion:—90 patients: indication for oral anticoagulant treatment other than atrial fibrillation (AF) (treatment or prophylaxis of venous thromboembolism, prophylaxis of acute coronary syndrome), previous diagnosis indicating valvular AF (mitral stenosis or artificial heart valves)—27 patients: no documented renal function

This retrospective study was performed using the Heart Center Leipzig database to identify consecutive patients with AF initiated on rivaroxaban between January 1, 2016 and January 31, 2017. Patients treated with rivaroxaban due to other conditions (treatment or prophylaxis of venous thromboembolism, prophylaxis of acute coronary syndrome), with valvular AF (mitral stenosis or artificial heart valves) or for whom the renal function was not documented, were not included (Figure 1).

Data were collected using a combination of patients' medical records and laboratory test results. Gathered data included sex, age, body mass index (BMI), prescribed rivaroxaban dose, renal function, further dosing‐relevant secondary diagnoses and medical history (including, eg, previous bleeding), CHA2DS2‐VASc (congestive heart failure, hypertension, age ≥ 75 years, diabetes mellitus, stroke/transient ischemic attack, vascular disease, age 65‐74 years, sex) score, HAS‐BLED (hypertension, abnormal renal and liver function, stroke, bleeding, labile international normalized ratios, elderly, and drugs or alcohol) score, pre‐ and co‐medication with antiplatelet drugs and NOACs. Transcatheter aortic valve implantation (TAVI), coronary stenting and other medical procedures which were performed over the last 6 months before choosing the dosing pattern and may influence the treatment were also included in the analysis.

### 2.2 Renal function and off‐label dosing

The main outcome was coherence or incoherence of the first rivaroxaban prescribing order with the package insert (PI) labeling. Patients with an eGFR ≥50 mL/min and a prescribed reduced dose of 15 mg q.d were categorized as potentially underdosed. An eGFR of 15 to 49 mL/min classified patients with prescribed dose of 20 mg q.d. as being potentially overdosed. Treatment was considered appropriate if it was consistent with the EMA‐guidelines. Furthermore, the physician letters were searched for reasons for the off‐label treatment.

### 2.3 Statistical analysis

All statistical analyses were conducted by using IBM SPSS Statistics 24.0 (Armonk, New York). Data are presented as mean ± SD, raw numbers and percentages. The cohort differences were assessed by statistical tests of significance (χ^2^ for categorical variables, *t* test for continuous variables). The identification of independent predictors of sub‐optimal rivaroxaban therapy was made by multivariate regression analysis, that included variables with a *P*‐value <.1 found in univariate analysis. A *P*‐value of <.05 was considered statistically significant.

## 3 RESULTS

A total of 378 patients with non‐valvular AF who were initiated on rivaroxaban were identified. Among 378 (100%) patients, the mean age was 66.5 ± 12.7 years, 265 (70.1%) male, and 113 (29.9%) female. The mean BMI was 28.6 ± 5.3 kg/m^2^. The eGFR had a mean of 72.9 ± 20.5 mL/min/1.73 m^2^. No patients with an eGFR <15 mL/min/1.73 m^2^ were found. The estimated risk for stroke, as summarized by the mean CHA2DS2‐VASc score was 3 ± 1.9 and the risk of bleeding measured by using the mean HAS‐BLED score was 1.9 ± 1.3. 19 (5.0%) patients had a bleeding event prior to the beginning of rivaroxaban therapy, most commonly a gastrointestinal one. The majority of patients (340; 89.9%) had not taken any oral anticoagulant previously. Overall 39 (10.3%) patients were on concomitant single antiplatelet therapy (SAPT) and only 19 (5.0%) on dual antiplatelet therapy (DAPT). All patients received the triple therapy within the intervention‐specific recommended time frame. Approximately, 17 (4.5%) patients received a TAVI, 70 (18.5%) ablation, 111 (29.4%) cardioversion, 24 (6.3%) pacemaker, and 31 (8.2%) a coronary intervention or stent within 6 months before rivaroxaban therapy was started. Almost three quarters of patients (283; 74.9%) had hypertension, 124 (32.8%) vascular disease, 112 (29.6%) heart failure, 94 (24.9%) diabetes mellitus, 27 (7.1%) experienced stroke or transient ischemic attack (TIA) (Table 1).

**Table 1:** Baseline characteristics values in mean ± SD or %

Characteristics	All patients (n = 378)	Patients with eGFR = 15–49 mL/min (n = 58)	Patients with eGFR ≥50 mL/min (n = 320)
Age, years	66.5 ± 12.7	76.2 ± 7.7	64.8 ± 12.6
≤65	157 (41.5)	5 (8.6)	152 (47.5)
64‐74	103 (27.2)	17 (29.3)	86 (26.9)
≥75	118 (31.2)	36 (62.1)	82 (25.6)
Sex			
Male	265 (70.1)	42 (72.4)	223 (69.7)
Female	113 (29.9)	16 (27.6)	97 (30.3)
Body mass index, kg/m^2^	28.6 ± 5.3	29 ± 5.8	28.5 ± 5.2
Underweight (<18.5)	1 (0.3)	1 (1.7)	0 (0.0)
Normal (18.5‐24,9)	78 (20.7)	11 (19.0)	67 (20.9)
Overweight (25‐29.9)	167 (44.4)	23 (39.7)	144 (45.0)
Obese (>30)	130 (34.6)	23 (39.7)	107 (33.4)
Rivaroxaban dose			
15 mg q.d.	96 (25.4)	47 (81.0)	49 (15.3)
20 mg q.d.	282 (74.6)	11 (19.0)	271 (84.7)
Creatinine, μmol/L	86.2 ± 23.5	121.4 ± 29	79.9 ± 15.4
eGFR, mL/min	72.9 (20.5)	39.7 ± 7.4	78.9 ± 15.8
15**‐**49 mL/min	58 (15.3)	58 (100)	0 (0.0)
≥50 mL/min	320 (84.7)	0 (0.0)	320 (100)
CHA2DS2‐VASc score	3 ± 1.9	4.4 ± 1.3	2.7 ± 1.8
0‐1	96 (25.4)	2 (3.4)	94 (29.4)
2‐3	128 (33.9)	14 (24.1)	114 (35.6)
≥4	154 (40.7)	42 (72.4)	112 (35.0)
HAS‐BLED score	1.9 ± 1.3	3.5 ± 0.8	1.6 ± 1.2
0‐2	260 (68.8)	4 (6.9)	256 (80.0)
≥3	118 (31.2)	54 (93.1)	64 (20.0)
Prior oral anticoagulant			
Phenprocoumon	28 (7.4)	9 (15.5)	19 (5.9)
Apixaban	6 (1.6)	2 (3.4)	4 (1.3)
Edoxaban	3 (0.8)	2 (3.4)	1 (0.3)
Dabigatran	1 (0.3)	1 (1.7)	0 (0.0)
None	340 (89.9)	44 (75.9)	296 (92.5)
Prior bleeding	19 (5.0)	6 (10.3)	13 (4.1)
Localization			
Gastrointestinal	8 (2.1)	1 (1.7)	7 (2.2)
Aquired bleeding disorders	6 (1.6)	2 (3.4)	4 (1.3)
Epistaxis	3 (0.8)	2 (3.4)	1 (0.3)
Intracranial	2 (0.5)	1 (1.7)	1 (0.3)
Antiplatet drug			
Clopidogrel	30 (7.9)	8 (13.8)	22 (6.9)
ASA + clopidogrel	17 (4.5)	2 (3.4)	15 (4.7)
ASA	9 (2.4)	2 (3.4)	7 (2.2)
ASA + tricagrelor	2 (0.5)	0 (0.0)	2 (0.6)
None	320 (84.7)	46 (79.3)	274 (85.6)
Interventions			
Cardioversion	111 (29.4)	16 (27.6)	95 (29.7)
Ablation	70 (18.5)	6 (10.3)	64 (20.0)
Stent/PTCA	31 (8.2)	6 (10.3)	25 (7.8)
Pacemaker	24 (6.3)	7 (12.1)	17 (5.3)
TAVI	17 (4.5)	5 (8.6)	12 (3.8)
Hypertension	283 (74.9)	55 (94.8)	228 (71.3)
Vascular diseases	124 (32.8)	30 (51.7)	94 (29.4)
Congestive heart failure	112 (29.6)	30 (51.7)	82 (25.6)
Diabetes mellitus	94 (24.9)	22 (37.9)	72 (22.5)
Stroke/TIA	27 (7.1)	6 (10.3)	21 (6.6)

Abbreviations: ASA, acetylsalicylic acid; CHA2DS2‐VASc, congestive heart failure, hypertension, age ≥ 75 years, diabetes mellitus, stroke/transient ischemic attack, vascular disease, age 65‐74 years, sex; eGFR, estimated glomerular filtration rate; HAS‐BLED, hypertension, abnormal renal and liver function, stroke, bleeding, labile international normalized ratios, elderly, and drugs or alcohol; TCA, percutaneous transluminal coronary angioplasty; TAVI, transcatheter aortic valve implantation; TIA, transient ischaemic attack.

In 282 (74.6%) patients, rivaroxaban was prescribed in a standard dose (20 mg q.d.) and in 96 (25.4%) in a reduced dose (15 mg q.d.). None of the patients received rivaroxaban in a 10 mg q.d. dose. A total of 317 (83.9%) patients were prescribed an appropriate dose [271 (71.7%): 20 mg q.d.; 46 (12.2%): 15 mg q.d.]. Fifty (13.2%) patients without renal impairment were initiated on a reduced dose (potentially underdosed) and 11 (2.9%) patients with renal impairment and indication for dose reduction, were prescribed the 20 mg dose (potentially overdosed). Due to its small size, we did not include the last group in the statistical analysis.

Potentially underdosed patients were younger, had lower serum creatinine level, and higher eGFR, lightly lower HAS‐BLED‐score, more frequent vascular disease and stent intervention and were on concomitant DAPT more frequently compared to patients receiving a reduced dose according to the PI.

Additionally, in potentially underdosed patients the eGFR was more often close to the cut‐off value compared with patients receiving doses consistent with the PI. In patients with no renal indication for dose reduction, those receiving a standard dose had better renal function than those receiving a reduced dose (mean eGFR 80.5 ± 15.5 mL/min/1.73 m^2^ vs 69.1 ± 15.3 mL/min/1.73 m^2^) (Table 2).

**Table 2:** Baseline characteristics according to dose groups, values in mean ± SD or %

Characteristics	Group 1	Group 2	Group 3	Group 2 vs Group 3
	20 mg correct (n = 271)	15 mg potentially underdosed (n = 50)	15 mg correct (n = 46)	*P*‐value
Age, years	63.2 ± 12.7	73.7 ± 8.7	77.1 ± 6.8	.035
≤65	143 (52.8)	9 (18.0)	3 (6.5)	
64‐74	72 (26.6)	14 (28.0)	12 (26.1)	
≥75	56 (20.7)	27 (54.0)	31 (67.4)	
Sex				
Male	191 (70.5)	33 (66.0)	34 (73.9)	.399
Female	80 (29.5)	17 (34.0)	12 (26.1)	.399
Body mass index, kg/m^2^	28.8 ± 5.4	27 ± 3.9	28.4 ± 5.2	.148
Creatinine, μmol/L	80.3 ± 14.8	78.3 ± 19.0	124.9 ± 29.9	<.0001
eGFR, mL/min	80.5 ± 15.5	69.1 ± 15.3	38.5 ± 7.6	<.0001
CHA2DS2‐VASc score	2.4 ± 1.7	4.5 ± 1.7	4.4 ± 1.4	.69
HAS‐BLED score	1.4 ± 1.0	3 ± 1.0	3.5 ± 0.8	.014
Prior OAC naive	251 (92.6)	45 (90.0)	35 (76.1)	.068
Prior OAC received	0 (0.0)	1 (2.0)	2 (4.3)	.068
Prior bleeding	5 (1.8)	8 (16.0)	5 (10.9)	.46
Antiplatelet drug				
SAPT	7 (2.6)	0 (0.0)	1 (2.2)	.099
DAPT	0 (0.0)	15 (30.0)	2 (4.3)	.0001
Interventions				
Ablation	62 (22.9)	2 (4.0)	5 (10.9)	.254
Cardioversion	88 (32.5)	7 (14.0)	11 (23.9)	.21
Stent/PTCA	2 (0.7)	24 (48.0)	5 (10.9)	<.0001
TAVI	1 (0.4)	11 (22.0)	5 (10.9)	.144
Pacemaker	13 (4.8)	4 (8.0)	6 (13.0)	.51
Congestive heart failure	54 (19.9)	28 (56.0)	23 (50.0)	.56
Hypertension	184 (67.9)	44 (88.0)	44 (95.7)	.27
Diabetes mellitus	60 (22.1)	12 (24.0)	17 (37.0)	.17
Stroke/TIA	12 (4.4)	10 (20.0)	4 (8.7)	.12
Vascular diseases	58 (21.4)	37 (74.0)	24 (52.2)	.026

Abbreviations: CHA2DS2‐VASc, congestive heart failure, hypertension, age ≥ 75 years, diabetes mellitus, stroke/transient ischemic attack, vascular disease, age 65‐74 years, sex; DAPT, dual antiplatelet therapy; eGFR, estimated glomerular filtration rate; OAC, oral anticoagulation; PTCA, percutaneous transluminal coronary angioplasty; SAPT, single antiplatelet therapy; TAVI, transcatheter aortic valve implantation; TIA, transient ischemic attack.

### 3.1 Predictors of rivaroxaban underdosage

In multivariate regression analysis, eGFR (OR 0.34, 95% CI 0.12‐0.95, *P* = .04) was the only independent predictor of rivaroxaban underdosage. Other factors which were included in the model [age (*P* = .73), concomitant dual antiplatelet therapy (*P* = .07), HAS‐BLED‐score (*P* = .27), vascular diseases (*P* = .84) and previous Stent/PTCA within the last 6 months prior to the start of rivaroxaban therapy (*P* = .09)] had no significant influence on off‐label dose reductions of rivaroxaban. There was no statistically significant effect of the concomitant dual antiplatelet therapy on the dose reduction, probably because of the inadequate amount of observational data. But there was a significant numerical difference observed between Groups 2 and 3. Whereas in the Group 3 only two patients (4.3% of 46) got a DAPT prescribed, in the Group 2 (potentially underdosed patients) there were 15 patients (30% of 50) on DAPT (Table 2).

### 3.2 Rationales for rivaroxaban underdosage as documented in medical records

In 33 (66.0% of 50) potentially underdosed patients, no reason for dose reduction was specified. In 17 (34.0% of 50) potentially underdosed patients, the reasons for underdosing, stated in the discharge letter, were simultaneous therapy with two antiplatelet drugs in 15 (88.0%) patients. Two (12.0%) patients had the dose prescribed due to previous severe gastrointestinal bleeding.

## 4 DISCUSSION

This study shows that in routine clinical practice rivaroxaban is often administered at an off‐label dose. Recent registry data suggest that off‐label dosing of NOACs is not uncommon.

In XANTUS (Xarelto for Prevention of Stroke in Patients with Atrial Fibrillation), the first international, prospective, observational study of patients with AF prescribed rivaroxaban, 15% of 3812 patients without renal impairment received the dose of 15 mg q.d., 36% of 640 patients with renal impairment and indication for dose reduction were prescribed the 20 mg dose.^8^ Compared to results in our study, the frequency of potentially underdosed rivaroxaban was almost equal (15.6% vs 15.0%), whereas the frequency of potentially overdosed rivaroxaban was clearly rarer (18.9% vs 36.0%). Nevertheless, the difference between both results might be caused by different sizes of patient populations. Whereas in XANTUS 640 patients were classified as having a renal impairment, in our study only 58 patients had a documented renal impairment.

In another large, clinical US‐study, the ORBIT‐AF II registry (Outcomes Registry for Better Informed Treatment of Atrial Fibrillation), out of 5738 AF‐patients treated with rivaroxaban, dabigatran or apixaban, 9.4% patients were potentially underdosed and 3.4% patients were potentially overdosed.^11^ Furthermore, out of all patients taking rivaroxaban, 8.0% were prescribed an inappropriately low dose and 4.8% were prescribed an inappropriately high dose. In comparison, the inappropriate dosing rate in our study was higher in potentially underdosed (13.2% vs 8.0%) and lower in potentially overdosed patients (2.9% vs 4.8%). The possible explanation for that could be different models of the study. We used the data from a specialized Heart Center and the ORBIT‐AF II used a nationally representative, more comprehensive sample of locations with cardiologists, as well as neurologists and care providers as prescribers. Additionally, the ORBIT‐AF II labeling of rivaroxaban is coherent with US FDA's guidelines which exclude a specific proposal of dose reduction for patients with severe renal dysfunction (eGFR of 15‐29 mL/min), in contrast with the EMA labeling guidelines. The ORBIT‐AF‐II does not include the prescribing habits of the medical personnel.

As mentioned above, the ORBIT‐AF II registry considered the dosing of patients being on rivaroxaban, dabigatran, and apixaban. In this study, apixaban had the highest inappropriate dosing rate (13.9%), followed by rivaroxaban (12.3%) and dabigatran (8.0%). Most inappropriately dosed patients were the ones who were underdosed. In comparison with patients taking rivaroxaban (8.0% being potentially underdosed and 4.8% being potentially overdosed), patients on dabigatran were prescribed an inappropriately low (7.5%) and high (0.5%) dose less often. On the other hand, apixaban was prescribed underdosed more frequently (11.8%) and overdosed more rarely (2.1%). However, there is a big disproportion between the numbers of patients receiving each medication. Rivaroxaban, with the number of 3078, was the most commonly prescribed NOAC in this study. 2235 patients were on apixaban and only 425 patients received dabigatran.

The main finding of another retrospective study performed in the Heart Center Leipzig in 2016—Initial apixaban dosing in patients with AF, was that apixaban was frequently dosed inappropriately in patients with AF, with underdosing being more common than overdosing.^12^ 569 patients with AF, initiated on apixaban, were found. In 301 (52.9%) patients, apixaban was prescribed in a standard dose [5 mg twice a day (b.i.d.)] and in 268 (47.1%) in a reduced dose (2.5 mg b.i.d.). Two‐hundred and sixty eight patients were administered a reduced dose, of whom 163 (28.6% of 569) were possibly underdosed and 102 (17.9% of 569) met the conditions for a dose reduction. A possible overdosing was observed in 13 cases (2.3%) only. Compared to the results in our study, the frequency of potentially underdosed patients was clearly rarer (13.2% vs 28.6%), whereas the frequency of potentially overdosed ones was almost equal (2.9% vs 2.3%). The difference of the off‐label underdosing might be caused by comparably more complicated guidelines for apixaban which require a dose reduction in case of at least two of these three conditions being met: serum creatinine ≥1.5 mg/dL, body weight ≤ 60 kg, and/or age ≥ 80 years.^13^

In multivariate regression analysis, we could prove that the decision for a lower than recommended dose of rivaroxaban is significantly associated with patients' eGFR, the same factor that is used as criterion for dose adjustment in the EMA‐approved PI of rivaroxaban. According to PI labeling, 20 mg q.d. are recommended as a standard dose in patients with eGFR ≥50 mL/min and a 15 mg q.d. as reduced dose in patients with renal impairment and eGFR of 15 to 49 mL/min.^5^ These recommendations were established in accordance with a pharmacokinetic model in which the creatinine clearance measurement showed a heightened rivaroxaban exposure in correlation with decreased renal functions. Serum rivaroxaban concentrations increased significantly with a creatinine clearance <50 mL/min.^6^, ^7^

We could see that physicians often decided for a reduced dose in patients with eGFR closer to the cut‐off value compared with patients receiving doses consistent with the PI. Among patients without a renal indication for dose reduction, the ones who received a reduced dose exhibited worse renal function than those who received a standard dose (mean eGFR 69.1 ± 15.3 mL/min/1.73 m^2^ vs 80.5 ± 15.5 mL/min/1.73 m^2^ vs) (Table 2). It leads us to believe that the off‐label dose reduction of rivaroxaban was caused mainly by eGFR being close to the cut‐off limit.

Furthermore, physician letters regularly do not include any justification for the use of an inadequate reduced dose. The reasoning given for this was concomitant antiplatelet therapy with two antiplatelet drugs and prior bleeding events. In our opinion, recording the reasons for inadequate dose reductions may be very helpful with physicians' applying of rivaroxaban's various doses in accordance with their individual indications and thereby diminish inappropriate dose reductions.

The use of antiplatelet therapy combined with NOACs has been prescribed in “2018 Joint European consensus document on the management of antithrombotic therapy in atrial fibrillation patients presenting with acute coronary syndrome and/or undergoing percutaneous cardiovascular interventions” with rivaroxaban 15 mg as a possible option combined with DAPT.^14^ The design and conclusions of the Pioneer‐AF‐PCI trial were used as a foundation of the proposed rivaroxaban 15 mg dosing method.^15^ So far, the strategy of rivaroxaban 20 mg, in patients with no renal impairment, with DAPT or SAPT is also supported by clinical guidelines. Our data show that the combination of DAPT with 15 mg rivaroxaban is very common since it was observed in almost one third of all potentially underdosed patients (30%; 15 of 50) in this study. It is highly arguable whether this dose was incorrect under these conditions. There were no patients on DAPT with prescribed standard rivaroxaban dose of 20 mg (Table 2). These lower doses of rivaroxaban may be caused by physicians' research‐proven fear of bleeding.^16^ Although all anticoagulant therapies are associated with some bleeding risk, this negative incident may be reduced by consistently using evidence‐based clinical evaluation scales, such as the HAS‐BLED‐score. In our study, among patients without renal indication for dose reduction, the HAS‐BLED score was higher in those treated with a reduced dose than in those treated with a standard dose (mean HAS‐BLED score 3 ± 1.0 vs 1.4 ± 1.0) (Table 2). The reason for that may be the physicians' apprehension about excessive bleeding. However, patients who tend to bleed more easily, generally are also at a higher risk of stroke. In fact, studies of physician attitudes show that physicians have a tendency to concentrate mainly on risk factors for bleeding rather than stroke, which changes the risk assessment in favor of bleeding and probably inadequately low doses of NOACs.^17^

The main concern by patients with AF is what influence does the off‐label dosing have on the effectiveness and safety of rivaroxaban. Currently, only few data exist on the effect of these clinical practices. In the ORBIT‐AF II registry, Steinberg et al analyzed rivaroxaban, dabigatran and apixaban all together and found that underdosing of NOACs is significantly associated with increased cardiovascular hospitalization and overdosing with increased risk of all‐cause mortality.^11^ In other large U.S. administrative database, Yao et al showed that underdosing of rivaroxaban is associated with no statistically significant trend toward lower risk of stroke.^10^ This association cannot be found in another database. Shrestha et al found no significant difference in stroke risk for both underdosed or overdosed patients compared with appropriately dosed patients taking NOACs for AF‐prevention. However, it was found that underdosed as well as overdosed patients have increased risk of bleeding compared with appropriately dosed ones.^9^ Hence, further research is required to prove the connection between off‐label dosing of rivaroxaban and patients' health. In daily practice only prescribing the right dose to the right patient can assure the achieving of the results that NOACs demonstrated in randomized clinical trials.

## 5 LIMITATIONS

This study has some limitations. It was a retrospective study and the data were from a single medical center. Additionally, not all concomitant medicines that might impact rivaroxaban treatment were collected. Last, no data was available, as to whether the dose was corrected and the association with clinical outcomes (eg, strokes, systemic embolism) was not reported.

## 6 CONCLUSIONS

In routine clinical practice, the prescribed rivaroxaban doses in patients with non‐valvular AF are often incoherent with EMA labeling. In this study, inappropriate rivaroxaban dosing occurred in patients with normal and insufficient renal function, with potential underdosing being more common (13.2% vs 2.9%). Inappropriate dose reduction was significantly associated with eGFR, the same factor that is used as a criterion for dose adjustment. Only few data exist on the effect of inappropriate rivaroxaban dosing in patients with AF and we believe that further research is required to prove the connection between off‐label dosing of rivaroxaban and patients' health. In daily practice, only prescribing the dose appropriately to the case can assure the achieving of the results that NOACs demonstrated in randomized clinical trials.
